# Electrochemical immunosensor based on gold-thionine for detection of subarachnoid hemorrhage biomarker

**DOI:** 10.3389/fbioe.2023.1153987

**Published:** 2023-03-08

**Authors:** Mengyue Wang, Feng Gao, Shoujie Ni, Yanan Song, Cai Wang, Qian Li, Peng Zhao

**Affiliations:** ^1^ The Second Affiliated Hospital, Shandong First Medical University & Shandong Academy of Medical Sciences, Taian, Shandong, China; ^2^ Qingdao Medical College of Qingdao University, Qingdao, Shandong, China

**Keywords:** THI, AuNP, immunosensor, SAH, IL-6

## Abstract

**Introduction:** In clinical work, the realization of an early diagnosis of Subarachnoid hemorrhage (SAH) is primarily based on conventional computed tomography (CT), MR angiography, transcranial Doppler (TCD) ultrasound, and neurological assessments. However, the association between imaging manifestations and clinical findings is insufficiently perfect, particularly in SAH patients in acute phases with a lower amount of blood. The establishment of a direct, rapid and ultra-sensitive detection method based on electrochemical biosensors has emerged as a new competitive challenge in disease biomarkers research.

**Methods:** In this study, a novel free-labeled electrochemical immunosensor for rapidly and sensitively detecting IL-6 in subarachnoid hemorrhage (SAH) blood has been developed using Au nanospheres-thionine composites (AuNPs/THI) as the interface modified on the electrode. Then, we detected IL-6 in blood samples from SAH patients by (enzyme-linked immunosorbent assay) ELISA and electrochemical immunosensor.

**Results:** Under the best conditions, the developed electrochemical immunosensor exhibited a wide linear range from 10^−2^ ng/mL to 10^2^ ng/mL with a low detection limit of 1.85 pg/mL. Furthermore, when the immunosensor was employed in the analysis of IL-6 in 100% serum, the results obtained by electrochemical immunoassay were consistent with those obtained by ELISA without suffering from other significant biological interference.

**Discussion:** The designed electrochemical immunosensor realizes the detection of IL-6 in actual serum samples with high accuracy and sensitivity, and could potentially become a promising technique for applications in the clinical diagnosis of SAH.

## Introduction

Subarachnoid hemorrhage (SAH) is an acute cerebrovascular event that leads to devastating effects on the central nervous system and is associated with a high mortality and morbidity rate. According to the report, 25%–40% of SAH patients will die within the first 30 days of their diagnosis, and approximately 40% will be permanently disabled, imposing a significant burden on individuals and society (Rodriguez-Rodriguez, et al., 2014). Aneurysmal subarachnoid hemorrhage accounts for approximately 80% of all SAH cases (Petridis et al., 2017). Early diagnosis of SAH and management of postoperative complications are major components of the clinical workload (Rass and Helbok, 2021). Rapid diagnosis of SAH in the acute phase plays a key role in decreasing complications following SAH caused by secondary neurological impairment, such as vasospasm, acute hydrocephalus, and rebleeding (Anetsberger et al., 2020; Ronne-Engstrom et al., 2014). Meanwhile, advancements in SAH treatment modalities and the application of dedicated neurointensive care units (NICUs) are directed at improving adverse outcomes resulting from secondary brain injury (Doerfler et al., 2018; Mackey et al., 2016; Molyneux et al., 2005). In clinical work, the realization of an early diagnosis of SAH is primarily based on conventional CT, MR angiography, transcranial Doppler (TCD) ultrasound, and neurological assessments. However, the association between imaging manifestations and clinical findings is insufficiently perfect, particularly in SAH patients in acute phases with a lower amount of blood (Vivancos et al., 2014). Similarly, it is also extremely difficult to rely on neurological assessment to make an accurate diagnosis of SAH, attributing it to the complex pathophysiological process and manifestation of the syndrome (Pan e tal., 2020). Significant evidence suggests that inflammatory responses are accompanied by the entire pathophysiology process, which provides a new clue for the development of SAH biomarkers (Hanafy et al., 2010; Wu et al., 2016). Inflammatory cytokines such as interleukin-1, interleukin-6 (IL-6), and tumor necrosis factor-alpha have been identified as potential biomarkers for SAH (Rodriguez-Rodriguez et al., 2014; Timmerman et al., 2015). IL-6 is a critical proinflammatory cytokine secreted by immune cells that is involved in inflammation, infections, metabolism, and tissue regeneration (Kao et al., 2015). Previous research revealed that elevated IL-6 levels in cerebrospinal fluid (CSF) and blood positively correlated with SAH-induced vasospasm and an unfavorable outcome (Sarrafzadeh et al., 2010).

At present, conventional methods utilized for the detection of IL-6 are Western blotting (WB) (Hu et al., 2020; Neri et al., 2011), enzyme-linked immunosorbent assay (ELISA) (Kuhle et al., 2016), and fluorescence analysis (Gunther et al., 2020). Although these measurement methods are able to achieve IL-6 detection to some extent, a variety of factors restrict further development, including complicated procedures requiring professional executors, low accuracy, time-consuming, and expensive instruments in the laboratory. The electrochemical immunoassay of specific antigen-antibody interactions has significant application in biomedical diagnostics (Hou et al., 2019; Takara et al., 2019). Electrochemical immunosensors, which are designed to be based on biosensing technology, have many significant advantages over conventional detection methods, such as simple operation, convenient carrying, and high sensitivity (Sun et al., 2020). According to the different interfacial features on the electrode, there are two types of electrochemical immunosensors that have been introduced for the sensitive detection of disease-related proteins, which are respectively called sandwich-type electrochemical immunosensors (Medetalibeyoglu et al., 2020) and label-free electrochemical immunosensors (Wang et al., 2018). Free-labeled electrochemical immunosensors are widely applied in the quantitative sensing of antigen, which is primarily determined by analyzing the physical changes of the complex modified on the electrode (Zeng et al., 2018; Zhang et al., 2017; Yin et al., 2018). In comparison with labeled electrochemical immunosensors, free-labeled electrochemical immunosensors are able to directly determine the various voltammetric current peaks induced by exclusive antigen-antibody recognition without modifying any tags, so that the electrochemical immunosensor can achieve highly efficient and specific detection for target molecules (Silva et al., 2013; Filik and Avan, 2019).

As one kind of highly sensitive electrochemical immunosensor, the key point of the response magnification strategy for free-labeled electrochemical immunosensors mostly depends on the nanomaterial modified on the electrode (Qian et al., 2021; Joshi and Kim, 2020). Various nanomaterials have been extensively employed in the format of the interface, which has the function of improving the stability and signal intensity of antigen-antibody recognition (Suresh et al., 2018). Among those nanomaterials, noble metal nanomaterials like AuNPs have many unique characteristics, including good electrical conductivity, high surface-to-volume ratios, and ideal biocompatibility capabilities (Lin et al., 2020; Lopez-Marzo et al., 2018). The thionine (THI) molecule has good electrochemical reversibility and stability and the ability to quickly transfer electrons due to the electroactive group contained in the molecular structure, making it a promising electrochemically active molecule (Yang et al., 2021; Dalkiran et al., 2020). Herein, encouraged by the superior characteristics of AuNPs and THI, we fabricated a novel electrochemical immunosensor based on the composites of AuNPs and THI capable of extensively improving the sensitivity and speed of the detection and applied it for the first time to the detection of IL-6 in SAH blood samples. Firstly, AuNPs were synthesized by the chemical reduction method and, by optimizing experimental conditions, the optimal size of AuNPs was obtained. A simple drop casting method was utilized for layer-by-layer modification of the AuNP, THI, and IL-6 antibodies in order to achieve the highest effective solid-liquid interface. During the experiment, fabrication parameters for the novel electrochemical immunosensor were also optimized, including antibody-antigen concentration, antibody-antigen binding duration and temperature. The novel electrochemical immunosensor with the lower LOD was successfully realized for the ultrasensitive detection of IL-6 in SAH, which provides new strategies for the clinical diagnosis of SAH.

## Materials and methods

### Chemicals and reagents

Hydrogen tetrachloroauric acid (HAuCl_4_·4H_2_O), ethanol, potassium hexacyanoferrate (II) (K_4_ [Fe(CN)_6_]) trihydrate, potassium hexacyanoferrate (III) (K_3_ [Fe(CN)_6_]), and bovine serum albumin (BSA) were brought from Shanghai Chemical Reagent Co., Ltd. China. Trisodium citrate dehydrate (CAS) and potassium chloride (KCl) were purchased from Kaitong Chemical Reagent Co., Ltd. (Tianjin, China). Thionin acetate (TIH) was purchased from Shanghai Maclean Biochemical Technology Co., Ltd. China. The IL-6 antibody and IL-6 antigen were acquired from Abcam. Phosphate-buffered saline (PBS) was bought from Solarbio Co., Ltd. (Beijing, China). All the reagents mentioned above were not further purified. The aqueous solutions used for the experiments were produced by a Millipore Direct-Q Water system (resistivity > 18 MΩ). All the experiments were conducted at room temperature.

### Apparatus

The morphologies of Au nanospheres were investigated using a Gemini SEM 300 scanning electron microscope. The UV–vis absorption spectra was detected using a Shimadzu UV-3600 plus spectrometer (Japan). The electrochemical measurements were conducted by a CHI660E electrochemical workstation (Shanghai Chenhua Co., China) with a conventional three-electrode setup consisting of a platinum wire as an auxiliary electrode, a saturated calomel electrode (SCE) as a reference electrode, and a bare or modified glassy carbon electrode as the working electrode.

### *S*ynthesis of AuNPs

AuNPs were synthesized using the previously reported method (Jiang et al., 2016). In brief, a solution of 0.01% HAuCl_4_ (20 mL) was poured into a beaker and heated. Until the solution was heated to boiling, 320 μL (1%) CAS solution was added into the solution. After stirring and heating for 30 min, the color of the mixture changed from transparent to gray and finally to burgundy. Then, the AuNPs solution was cooled to room temperature for later use.

### Fabrication of the electrochemical immunosensor

The drop casting method employed to modify electrodes was the same as that previously reported (Antuna-Jimenez et al., 2020). Firstly, the glassy carbon electrode was polished to a mirror finish with 0.05 m alumina slurries and then thoroughly washed with ethanol and ultrapure water in turn. The electrodes were dried in nitrogen gas after being cleaned with hydroxyl groups. After finishing cleaning and drying of the electrode, a drop of 5 μL as-prepared AuNPs solution was dipped onto the mirror of the cleaned electrodes to react with inorganic-OH on the electrode surface. Subsequently, the electrodes modified with AuNPs were dried in an oven at 37°C. Following the immobilization of AuNPs, the TIH was modified on the surface of AuNPs by dipping 5 μL (1 M) THI on the electrodes and dried again in a 37°C oven. Then, 4 μL of IL-6 antibody solution was dipped onto the Au/THI electrode and incubated at 37°C for 30 min. Using the same method, the anti-IL-6/Au/THI electrode was blocked to prevent non-specific adsorption using 4 μL of PBS buffer containing 1% BSA. After blocking the surface active sites, the electrochemical immunosensor synthesis was completed and stored at 4°C. When testing the sample, 5 μL of the liquid sample was loaded onto the electrochemical immunosensor. After 40 min of co-incubation at 30°C, the obtained electrode was immersed in a 5 mM K_3_ [Fe(CN)_6_]/K_4_ [Fe(CN)_6_] (1:1) solution that contains 0.1 M KCl for electrochemical measurements.

### Collection of human blood samples

Human blood samples were acquired from the affiliated hospital of Shandong First Medical University. The study protocol was approved by the ethical review board of the affiliated hospital of Shandong First Medical University. Consents were obtained after fully informing the patients about the study procedure. Whole blood was collected in a 5 mL Eppendorf tube containing EDTA and then centrifuged at 5,000 rpm for 10 min to separate serum. Prior to measurements, the obtained serum was collected and stored at −80°C.

### Experimental characterization

Cyclic voltammetry (CV), electrochemical impedance spectroscopic (EIS), and differential pulse voltammetry (DPV) techniques were employed for the characterization of the detection process. CV measurements were performed with a scan rate of 100 mV s^-1^ and a step size of 10 mV, and EIS measurements were carried out at 10,000 Hz of initial frequency and 0.01 Hz of final frequency in 12 points. DPV (potential range from −0.2–0.6 V, amplitude: 0.05 V, pulse width: 0.06 s) measurements were employed during the experiment. All characterization experiments were performed at room temperature.

## Results and discussion

### Characterization of AuNPs and AuNPs/THI/anti-IL-6/BSA

**FIGURE 1:**
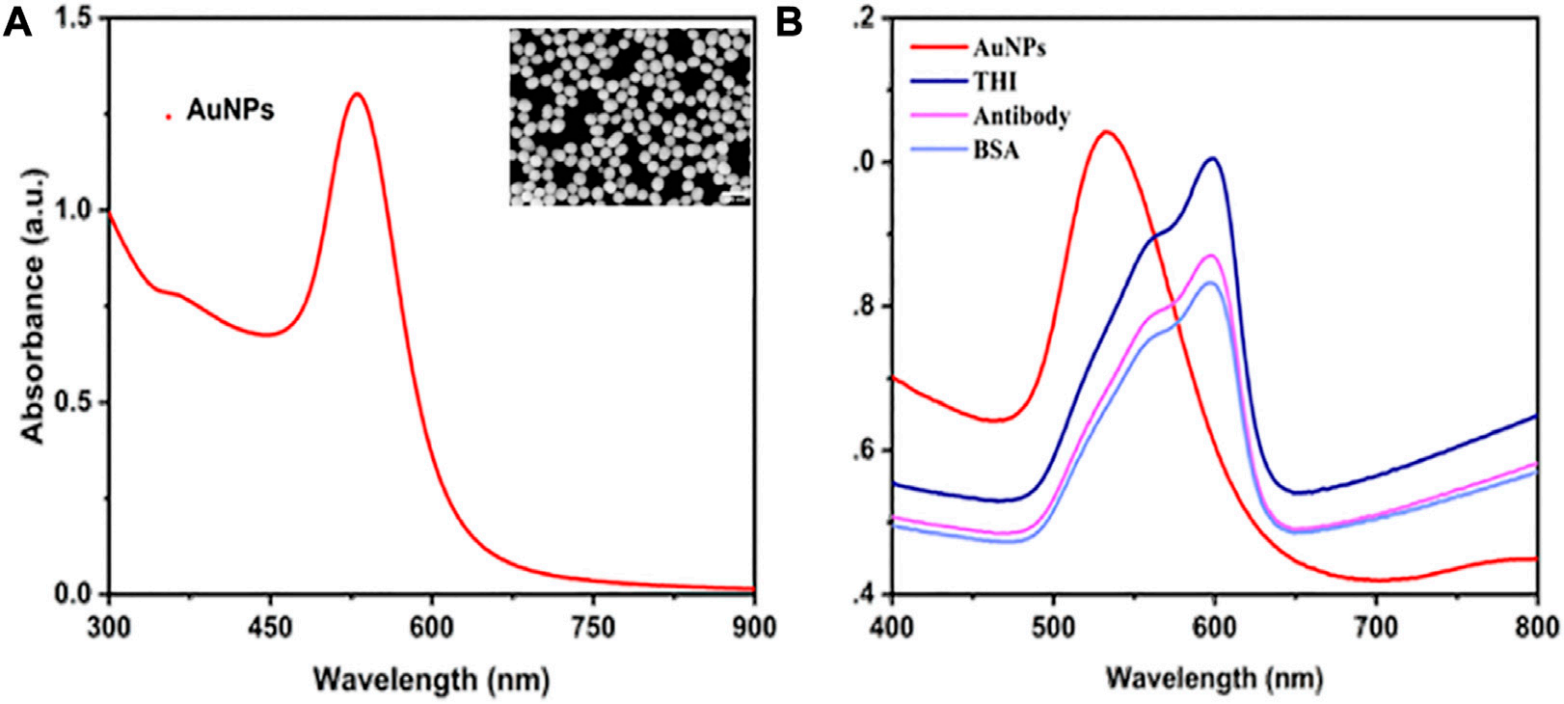
Characterization of AuNPs and probes. **(A)** UV-Vis absorbance spectrum of AuNPs. Inset: SEM image of AuNPs, scale bar = 400 nm. **(B)** UV-visible spectrum of step-by-step fabrication of the interface, including AuNPs, AuNPs modified with THI, AuNPs/THI modified with antibody, and AuNPs/THI/Antibody modified with BSA.

The morphology of AuNPs and their UV–Vis absorption spectra were investigated using SEM and UV-Vis, respectively. In Figure 1A, it was shown that there are characterized peaks of AuNPs that were recorded at around 532 nm. In the SEM image of AuNPs, all the AuNPs displayed a typical spherical structure with a uniform size, and the diameter of each AuNP was about 45 nm. The above results demonstrated that 45 nm of AuNPs were successfully synthesized. The detection probes for electrochemical immunosensors were created by modifying the anti-IL-6 on the surface of AuNPs/THI compounds and blocking them using BSA molecules. The UV-Vis-NIR spectra were used to record the probe modification process. As shown in Figure 1B, when the compounds of AuNPs/THI were formed, there was a slight reduction in the intensity of the absorption peak and a distinct red-shift of the maximum peak from 532 nm to 597 nm. The maximum peak was obviously reduced again after being coupled with ani-IL-6 onto the surface of the compounds of AuNPs/THI. Moreover, the surface Plasmon resonance band further decreased, resulting from the blocking process using the protective agents of BSA.

### Characterization of the manufacturing process of the electrochemical immunosensor

**SCHEME 1:**
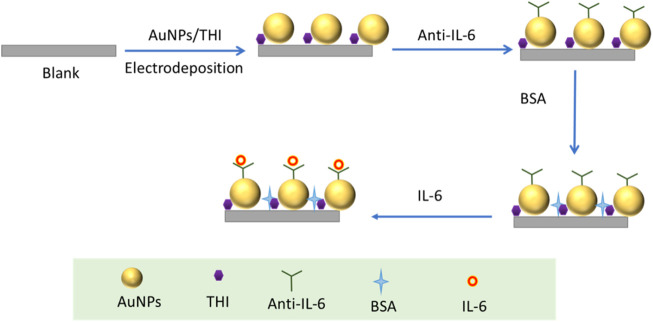
Fabrication process of the developed electrochemical immunosensor.

**FIGURE 2:**
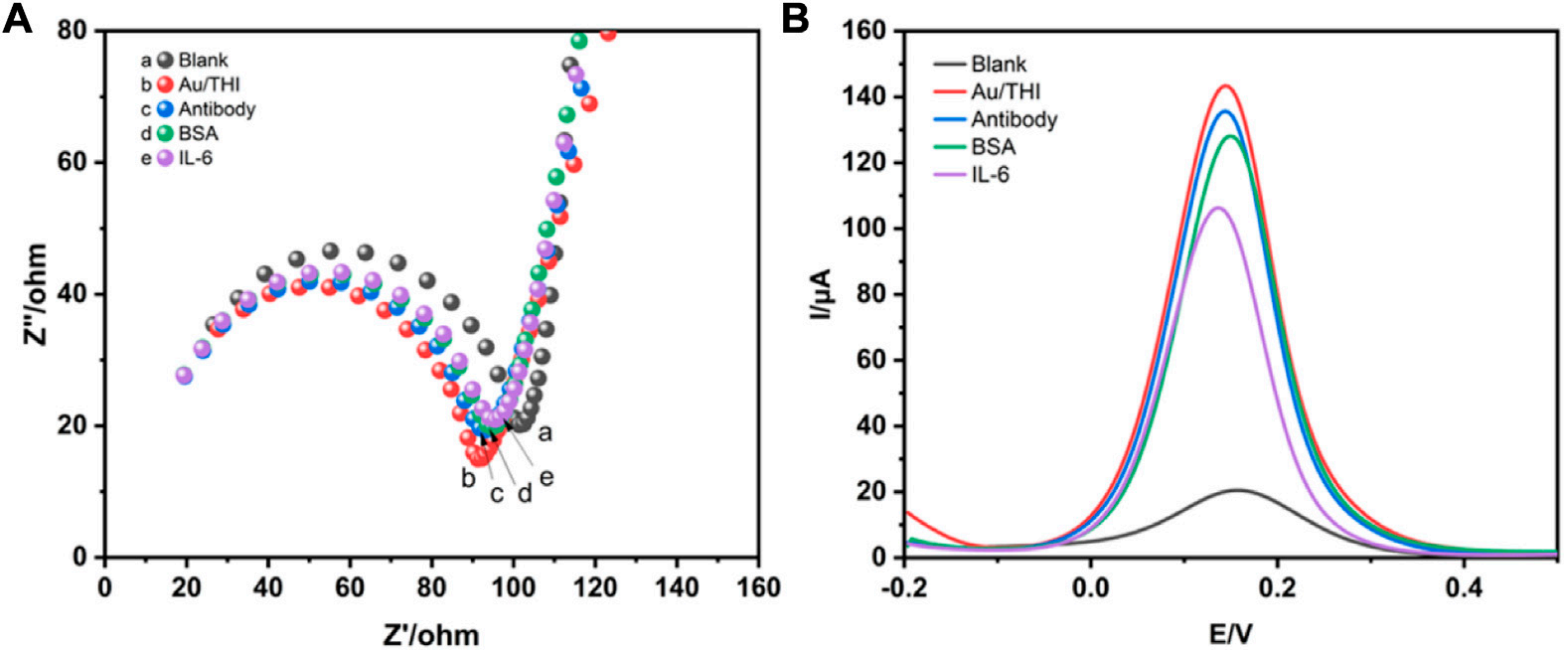
Characterization of immunosensors for preparation processes by EIS and DPV techniques. **(A)** The Nyquist impedance spectra of blank electrode (a), AuNPs/THI (b), AuNPs/THI/Antibody (c), AuNPs/THI/Antibody/BSA (d), AuNPs/THI/Antibody/BSA/IL-6 (e). **(B)** DPV for step-by-step fabrication of the developed electrochemical immunosensors.

The detailed manufacturing process of the developed electrochemical immunosensor was illustrated in Scheme 1, which recoded each stage of electrode surface modification with a modification mechanism, followed by the bare electrode; the composite of THI and AuNPs immobilized electrode; the electrode surface modified with the IL-6 antibody; blocking the remaining active site of the surface with 1% BSA; and conjugation with IL-6 on the interface of the electrode. To characterize the stepwise fabrication process of immunosensor, EIS and DPV techniques were utilized. As can be seen in Figure 2A, a nyquist plot was recorded to illustrate the variation of electron transfer resistance (Rct) at each modification step, which consists of a small semicircle and a linear part. The diameter of the semicircle represents Rct. When the blank electrode was modified with a composite of THI and AuNPs, the semicircle diameter of the THI/Au electrode decreased in the Nyquist plot owing to the composite’s high conductivity. Rct increased after adding IL-6 antibody to the composite surface, indicating the successful covalent binding of IL-6 antibody. When the BSA was used to block the remaining active ends of the IL-6 antibody, the Rct increased due to the BSA’s less conductive nature. During the final fabrication step of conjugation with IL-6, the impedance spectra also showed an increase of Rct of the Nyquist plot. This increase indicated that successful binding of antigen and antibody by IL-6 further blocked the electron transfer to the interface on the immunosensor. The results for EIS measurements indicated that the immunosensor was prepared successfully.

DPV technology is commonly used to monitor the immunosensor fabrication process and its performance changes. Figure 2B shows the DPV of different modified electrodes. Due to the high conductivity of the complex of THI and AuNPs, the DPV peaks of the THI/Au electrode increased as compared to the bare electrode. When an IL-6 antibody was conjugated on the surface of the THI/Au electrode, the DPV peaks were lowered. Likewise, the same change occurred with the addition of BSA to block the non-specific binding site of the IL-6 antibody. After finishing the last step for the fabrication of the immunosensor, the DPV peak further decreased. Furthermore, the results obtained from the DPV measurements were consistent with those from the EIS measurements. Based on the above results, the successful construction of an electrochemical immunosensor was further confirmed.

### Electrochemical mechanisms of the electrochemical immunosensor

**FIGURE 3:**
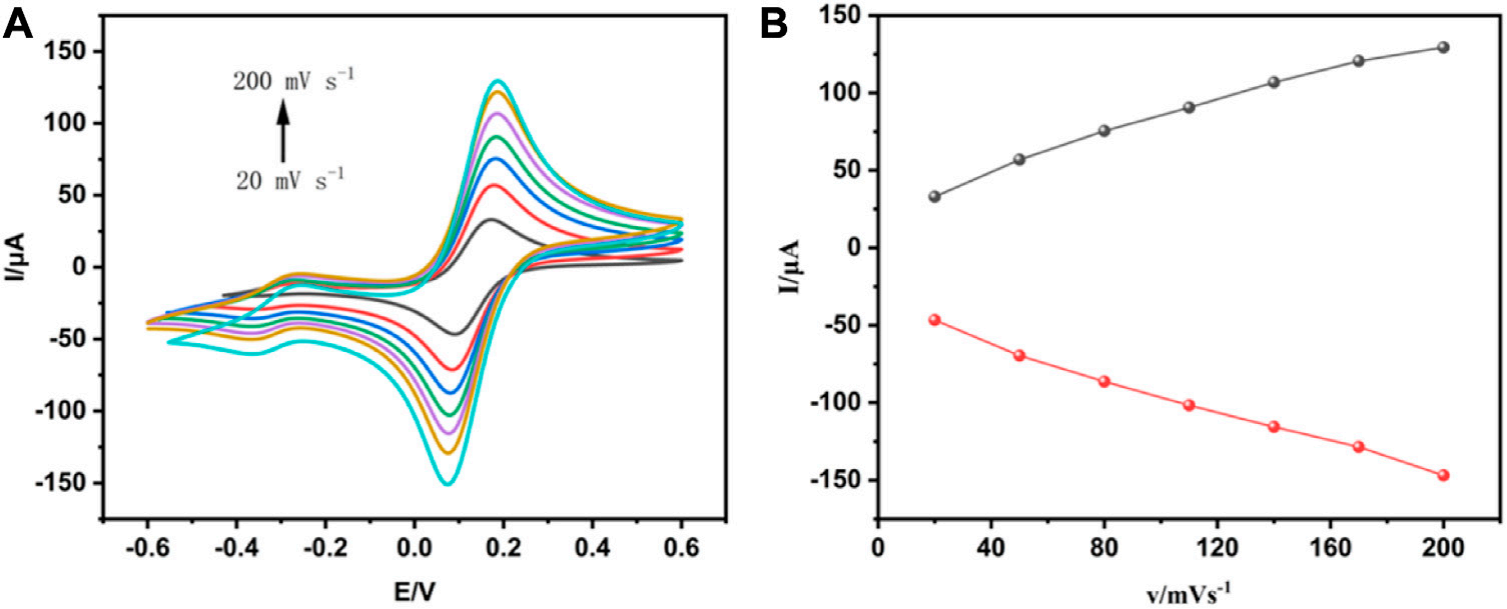
Analysis of the interfacial characteristics of modified electrode surfaces using CV techniques. **(A)** The cyclic voltammogram of the electrochemical immunosensor obtained at different scan rates. **(B)** The relationship between the redox peak current and the scan rate.

To further study the electrochemical mechanisms of the electrochemical immunosensor, the CV technique was used to assay the interface features of modified electrode surfaces at a scan rate range from 20–200 mV s^-1^. It was shown in Figure 3A that the redox peaks obtained from the anode and cathode increased successively as the scan rate increased. Moreover, there was a good linear relationship between peak current and scan rate, as seen in Figure 3B. This result indicated that the redox reaction on the electrode surface was controlled by the separation and binding of electroactive groups of THI. In addition, the catalytic performance of interface reduction was mainly based on the active sites in the electroactive area. The catalytic performance improved as the number of active sites increased.

### Optimization studies of electrochemical immunosensor

**FIGURE 4:**
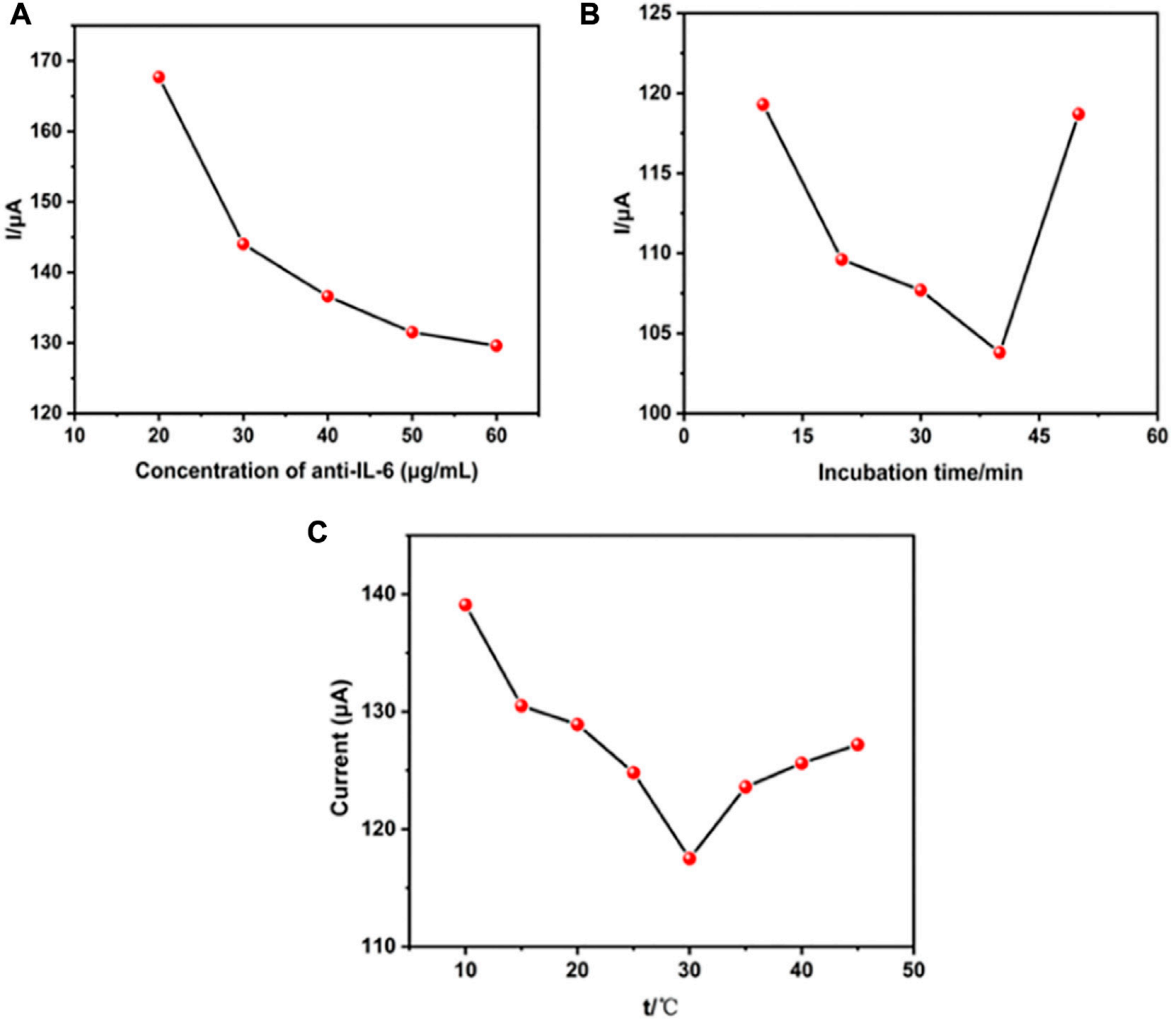
Effect of the experimental parameters. **(A)** The concentration of anti-IL-6 on the electrochemical immunosensor, **(B)** the incubation time and **(C)** incubation temperature.

To develop a sensitive and stable electrochemical immunosensor, the experimental parameters, including antibody concentrations and incubation duration of antibodies and antigens as well as the incubation temperature that significantly impact the immunosensor response, were optimized. Figure 4A shows the effect of IL-6 antibody concentrations on the fabrication of immunosensors. Among varied concentrations of IL-6 antibody from 20 μg/mL to 60 μg/mL, the best response was at concentrations of 40 μg/mL, in which the peak current remained practically constant. This phenomenon was due to antibody-linked Au/THI reaching saturation. Figure 4B shows the other optimization parameter process of antibody and antigen incubation duration. The peak current decreased with the incubation time because of the conjugation of antibody and antigen. However, this trend changed when the incubation time exceeded 40 min, which was attributed to the optimum interaction period of antibody and antigen. Another key parameter is the temperature at which the antigen-antibody reaction occurs. As shown in Figure 4C, an optimal antigen-antibody binding response was obtained by incubating the immunosensor with IL-6 antigen at 30°C. This was because an optimum reaction temperature at 30°C led to high reactivity for binding between the antigen and the detection antibody.

### Analytical characterization of electrochemical immunosensor

**FIGURE 5:**
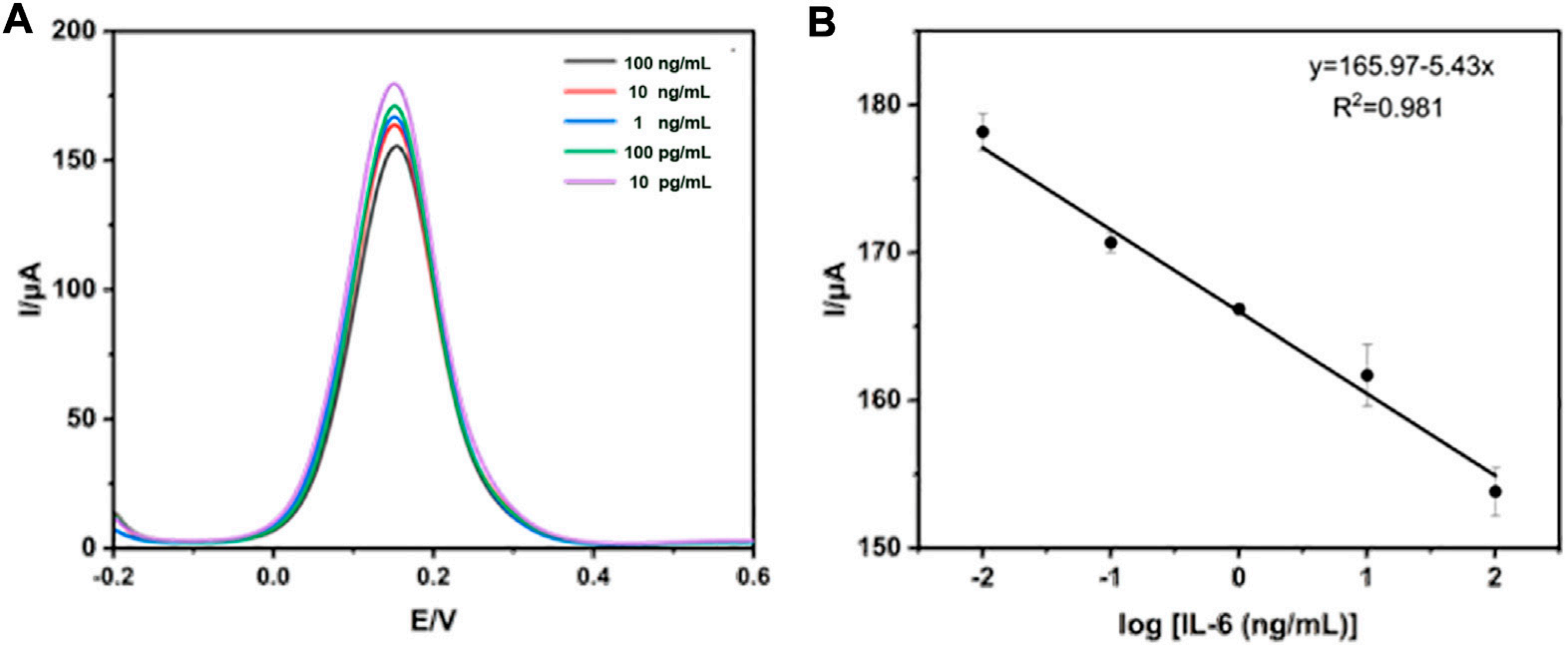
Detection of standard IL-6 using DPV technology. **(A)** The measurement of standard IL-6 ranged from 10^–2^–10^2^ ng/mL **(B)** The calibration curve of the electrochemical immunosensor.

To investigate the analytical performance of the constructed electrochemical immunosensor, DPV technology was used for the analysis of various concentrations of standard IL-6, ranging from 10^–2^–10^2^ ng/mL spiked into PBS under optimal conditions. As is well known, the mechanism of DPV technology for quantitative detection of antibody concentration is mainly based on the interface formed on the electrode surface, which is capable of blocking electron exchange. Once the amount of antigen-antibody binding increases, the conductivity of the interface will also gradually decrease in proportion, allowing for the quantification of IL-6. As can be seen in Figure 5A, the current peak obtained by detecting the interface modified with a standard sample of IL-6 decreased with the corresponding increase in the concentration of standard IL-6. Figure 5B exhibited a good linear relationship between the response values of DPV and the logarithm of the IL-6 concentration. The linear equation is I (A) = −5.43 ln CIL-6 (pg/mL) + 165.97, with a high linearly dependent coefficient (R^2^ = 0.981) and a detection limit (n = 3) of 1.85 pg/mL.

### Detection of IL-6 in human blood samples

To evaluate the practical feasibility and applicability of the proposed immunosensor, the diluted clinical serum sample as an antigen was utilized for DPV measurement, and the real concentration of IL-6 in the blood was calculated by the calibration curve obtained from Figure 5B. As shown in Table 1, the data obtained from ELISA to detect IL-6 in several SAH patients’ serum samples were compared with that from the constructed immunosensor. It was found that the results monitored by the proposed immunosensor assay were exactly consistent with those obtained through the traditional enzyme-linked immunosorbent assay (ELISA), proving that this electrochemical immunosensor is capable of being applied for the clinical detection of real blood samples.

**TABLE 1:** Comparative evaluation of the immunosensor with ELISA.

Sample	IL-6 (ng/mL)	
	ELISA	Immunosensor
NO.1	4.6	4.8
NO.2	7.4	1.5
NO.3	8.6	8.4
NO.4	10.2	10.3
NO.5	14.6	14.5

### The specificity and stability of the electrochemical immunosensor

**FIGURE 6:**
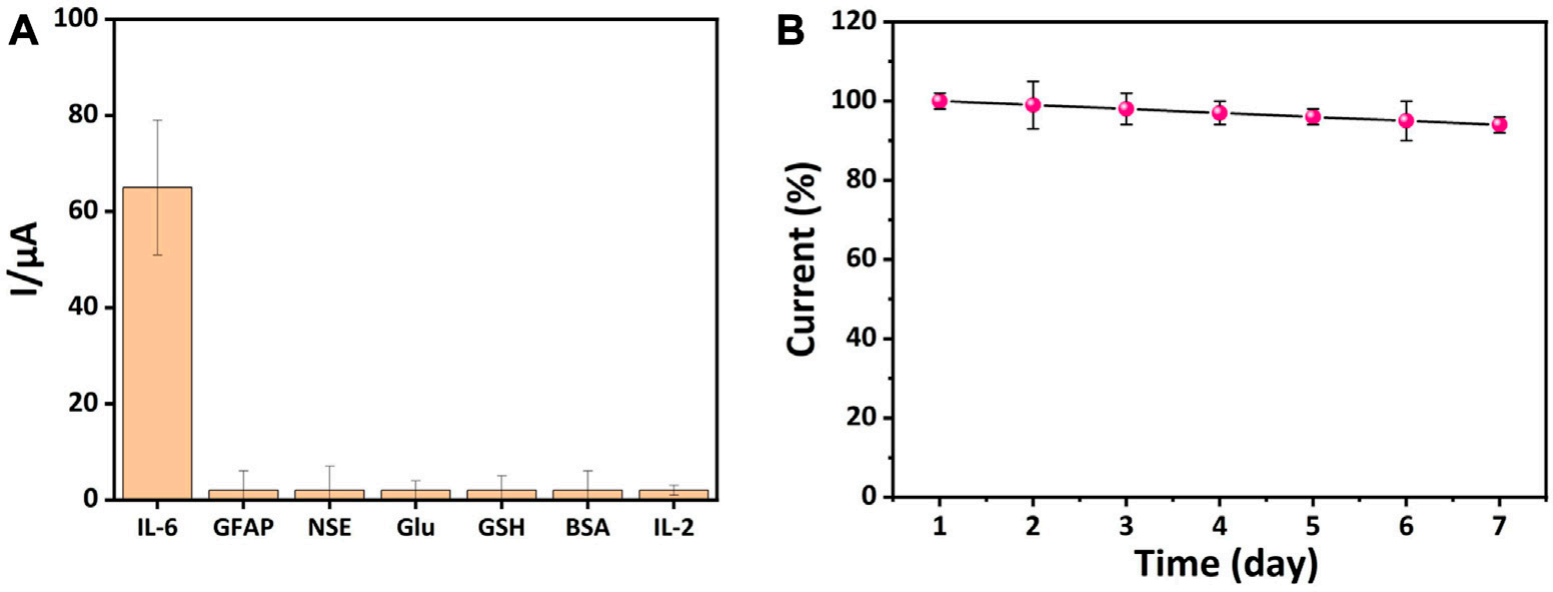
Detection of specificity and stability of electrochemical immunosensors. **(A)** The specificity of the constructed immunosensor towards GFAP, NSE, Glu, GSH, BSA, and IL-2 (The concentration of IL-6 was 10 pg/mL, and the interferents were 1 ng/mL). **(B)** The stability of the constructed immunosensor (n = 3).

For the analysis of specific biological components in complex biological products, the specificity and stability of the electrochemical immunosensor are extremely important. In order to assess the performance of the developed electrochemical immunosensor for detecting target analytes in complex biological products, the comparison experiment was performed using GFAP, NSE, Glu, GSH, BSA, and IL-2 as interferents. As shown in Figure 6A, the intensity of the current signals obtained from the IL-6 was much higher than that obtained from other interferents. To investigate whether the test performance of the developed electrochemical immunosensor was affected by time. The prepared electrochemical immunosensor was stored at 4°C and then evaluated each day. It was observed in Figure 6B that the current of the developed electrochemical immunosensor was slightly decreased with time as it was detected for seven consecutive days, which suggests that the electrochemical immunosensor possesses excellent performance stability. All the above results prove that the developed immunosensor is able to be utilized for practical measurement.

## Conclusion

In this paper, a novel free-labeled electrochemical immunosensor based on an AuNPs/THI complex-modified electrode has been successfully developed and demonstrated. The use of AuNPs/THI as the substrate for biological molecule immobilization not only significantly increased the specific surface area for antibody immobilization and modification but also dramatically facilitated electron transport and electrochemical signal enhancement. Using the layer-to-layer assembly method, a favorable platform on the electrode with high biocompatibility and electrical conductivity was successfully constructed. Further optimizing experimental conditions, the designed electrochemical immunosensor exhibited a highly sensitive detection performance, which was demonstrated to have a low detection limit of 1.85 pg/mL and a wide linear range of 10^–2^ ng/mL to 10^2^ ng/mL. Moreover, the designed electrochemical immunosensor realizes the detection of IL-6 in actual serum samples, and the result demonstrated a good stability and reproducibility as well as selectivity of the electrochemical immunosensor in the practical application. Thus, the designed electrochemical immunosensor could potentially become a promising technique for applications in the clinical diagnosis of SAH. Likewise, it could be extend for other various human diseases.

## Data Availability

The original contributions presented in the study are included in the article/supplementary material, further inquiries can be directed to the corresponding author.
